# The Relationship Between Behavioral Inhibition and Behavioral Activation Systems, Impulsiveness, and Internet Gaming Disorder Among Students of Different Ages

**DOI:** 10.3389/fpsyt.2020.560142

**Published:** 2021-01-12

**Authors:** Hui Xiang, Xiaolin Tian, Yu Zhou, Jieyu Chen, Marc N. Potenza, Qun Zhang

**Affiliations:** ^1^Department of Psychiatry, Guizhou Provincial People's Hospital, Guiyang, China; ^2^Department of Psychiatry, Yale University School of Medicine, New Haven, CT, United States; ^3^Child Study Center, Yale University School of Medicine, New Haven, CT, United States; ^4^Department of Neurobiology, Yale University School of Medicine, New Haven, CT, United States; ^5^Department of Psychology, Guizhou Normal University, Guiyang, China

**Keywords:** students with IGD, BIS, BAS, impulsivity, different grades

## Abstract

** Background and Aims:** Internet Gaming Disorder (IGD), which is an underlying symptom classified as a behavioral addiction disorder, has many related social problems that have garnered the bulk of attention in recent research. However, its psychological/behavioral mechanism is still unclear. The purpose of the current study was to explore the relationship between IGD and impulsivity which is regulated by behavioral inhibition and activation among different age groups.

**Methods:** A total of 1,525 students completed Young's Internet Addiction Test (IAT), Online Game Addiction Questionnaire, the Behavioral Inhibition System/Behavioral Activation System (BIS/BAS) scales, and the Barratt Impulsiveness Scale-11 (BIS-11).

**Results:** (1) The prevalence of IGD in different ages students was 10.3%. (2) BIS, BAS-Fun seeking (BAS-F), and BIS-11 scores indicated that there were significant differences between IGD and non-IGD individuals. (3) BIS showed significant differences in different grades (i.e., between undergraduates and middle school students with IGD; *F* = 3.10, *p* < 0.05). (4) The IAT scores demonstrated a significant positive correlation with BIS scores (*r* = 0.375, *p* < 0.05) among undergraduates with IGD; IAT scores of high school students with IGD were negatively correlated with BAS-Reward (BAS-R) (*r* = −0.28, *p* < 0.05).5)BIS and BIS-11 together could explain 14.9% variance of IGD among the college group; BAS-R and BAS-F together could explain 16.7% variance of IGD among the high school group (*p* < 0.05).

**Conclusions:** The relationships between IGD and BIS, BAS-F, BAS-D, BIS-11 scores differed due to the age group of individuals tested.

## Introduction

The “45th China Internet Development Statistics Report” which was published by the China Internet Network Information Center (1), showed that mobile Internet users account for an absolute majority of Internet users, accounting for 99.3 percent, while mobile Internet game users account for 59 percent of mobile Internet users. With the rapid development of society and widespread access to free public Wi-Fi and the ubiquity of smart phones, online games have become an important part of the private lives of college, high school, and middle school students. However, online gaming is a double-edged sword, which not only brings joy but may also make gamers unable to extricate themselves from the virtual world, causing harm to their physical and mental health. According to statistics, Internet addiction (IA) was the most common addiction type among Asian students, with the majority of IA being reported as online gaming addiction. This disorder can harm to the community, families, and individuals and is especially detrimental to the physical and mental health of students (2–4). The Diagnostic and Statistical Manual of Mental Disorders, fifth edition (DSM-5) (5), incorporated Internet Game Disorder (IGD) into the category of psychiatric disorders that require further study, encouraging more IGD studies to be conducted.

Addictive behavior has often been explained by Gray's reinforcement sensitivity theory (RST) (6), which refers to an individual's sense of reward and punishment. Additionally, Gray argues that there are individual differences in sensitivity, and that these differences stemmed from two basic brain systems that control human behavior: the Behavioral Inhibition System (BIS) and the Behavioral Activate/ Approach system (BAS). The BIS is sensitive to punishment, novelty, and negative emotions, which may be related to activation within the hippocampus and the amygdala (7). Conversely, the BAS is sensitive to rewards and is thought to be related to positive emotions, such as happiness, which may be related to the striatum (8). The current assessment tool of the RST are BIS/BAS Scales (9), which includes the BIS and BAS scales. The BAS scale is divided into the BAS-Reward (BAS-R), BAS-Drive (BAS-D), and BAS-Fun seeking (BAS-F) subscales. Studies have shown that BAS scores are important predictors of drug abuse and alcohol addiction (10, 11). However, it is possible that IA could have a different pathogenesis than other dependences. Researchers believe that IA is more likely to be associated with BIS rather than BAS (12). For instance, in a study of college students, BIS and BAS-F scores were higher in students with IA than in students with alcohol dependence (13). Park (14) found that BIS and BAS-F scores independently predicted IA behavior in the adolescents. Yet, more recent studies have yielded inconsistent results. Specifically, a separate adolescent IA study found that elevated BAS and BAS-F scores were high-risk factors of IA (15). Furthermore, another study showed that BAS-D, BAS-R and impulsivity increased the risk of smartphone addiction (16). Given these findings, it is possible that the purpose of surfing the internet and ages have not been distinguished by previous studies. Therefore, the relationship between BIS/BAS and IA was inconsistent due to different these factors (Note: IA include IGD here).

The core symptoms of IGD is that patients are of the direct impairment of their social functioning due to online gaming but are unable to control their game-playing behavior. This would suggest an impulse control disorder that does not involve drug or alcohol (17). Studies have shown that there is a significant positive correlation between IA and impulsivity (18, 19), that can predict IA (20), and that 90% of IA falls under the classification of IGD. Impulsiveness is generally considered to be correlated with BAS (21). Slessarev et al. believed that negative emotions could regulate the relationship between BIS (Behavioral Inhibition System) and self-control (22).

The purpose of this study was to explore the relevance of RST and impulsivity in students with IGD in different age groups. This was done in an effort to determine psychological behavior mechanism underlying IGD, which would provide a theoretical basis for future psychological interventions. We hypothesized that impulsivity is a susceptibility factor for IGD in different age groups, and RST system plays a synergistic role.

## Methods

### Participant Selection

Data was collected using questionnaire surveys administered by two psychiatric postgraduates from September 10th to December 30th 2016. Participants were 1,600 students, aged 12–26 years, from Guizhou University of Technology,Guizhou Medical University, Guiyang Vocational and Technical College, Guiyang 31st Middle School, Guiyang 34th Middle School, and Guiyang 39th Middle School, and were chosen via stratified random cluster sampling. A total of 1,525 valid questionnaires were collected, representing a 95.3% response rate. The sample was composed of 878 males (57.6%) and 647 females (42.4%). Data was split into three categories: 794 college students, 316 high school students, and 415 middle school students.

The criteria for being placed in the IGD group were (1) IAT score> 50 points; (2) the main purpose of the Internet was for playing online games; (3) the average daily online game time was 4–6 h for more than a year. (4) Diagnostic of IGD. Students who meet the above criteria were diagnosed with IGD by attending psychiatrists in accordance with the diagnostic criteria for IGD in DSM-5 (5). We must also exclude the following rules: (1) Severe mental disorder, such as schizophrenia and major depression disorder; (2) severe cognitive dysfunction.

### Instruments

#### Young's Internet Addiction Test (IAT) (23)

The IAT is composed of 20 items rated on a 5-point scale, (1 = *very rarely*, 5 = *very frequently*). All item scores are summed to obtain an IAT total score. A total of 50 points and below indicates no problem with IA; 50–79 indicates mild IA; 80–100 indicates severe IA.

#### Self-Made Game Questionnaire

The questionnaire contains three questions: (1) What is the main purpose of your Internet use? (2) Which of the following do you spend the most time on? (A. Game, B. Other), and (3) How long have you played games each day over the past year? If the answers to the first two questions were “game,” the time spent was 4–6 h/day, the subject's IAT score> 50 points, and the individual was classified as exhibiting online game addiction.

#### BIS/BAS Scales (24)

The 24 item BIS/BAS Scales which were compiled by Carver and White (9), were translated into Chinese and tested reliability and validity by our preliminary study. The scales have demonstrated satisfactory reliability and validity. Our revised BIS/BAS scale was composed of four dimensions, the total Cronbach's α coefficient of the scales was 0.80, with each Cronbach's α coefficient of the BIS (5 items), BAS-R (6 items), BAS-D (4 items), BAS-F (3 items) was 0.67,0.77,0.68 and 0.59, respectively. The Chinese edition of BIS/BAS Scales consists of 18 items and 4 fillers rated on a 4-point Likert scale from “*totally agree*” to “*totally disagree*.”

#### Barratt Impulse Scale 11th Edition (BIS-11)

The Yang Huiqin revised BIS-11 (25) was used. The scale contains 30 questions, and Cronbach's α was 0.8.

### Statistical Analysis

All the data was entered into an Excel database. A *t*-test was used to examine the differences in BIS/BAS and BIS-11 among individuals with and without IGD. In students with IGD, an ANOVA was used to examine the differences of BIS/BAS and BIS-11 among middle school students, high school students, and college students. All comparison tests were two-tailed. The relationship between IAT scores and scores of BIS, BAS-R, BAS-D, BAS-F, and BIS-11 was analyzed by Pearson correlation analysis. Regression analysis was conducted to test the roles of BIS-11, BIS, and all of the BAS-subscales as predictors of IAT scores. All statistical analyses were conducted using IBM SPSS Statistics for Windows Version 22.

## Results

### The Prevalence of IGD in Middle School Student and College Student

Utilizing criteria of IGD, 157 participants were classified as having IGD, accounting for 10.3% of the total. Of these, 66 were college students, 48 were high school students, and 43 were middle school students, aged 12–22 years.

### Comparison of BIS/BAS and BIS-11 Between Students With IGD and Without IGD

Table 1 shows that there were significant differences in BIS, BAS-F, and BIS-11 between students with and without IGD (*p* < 0.001), with scores of students with IGD being greater in the BIS, BAS-F and BIS-11.

**Table 1:** BIS/BAS and BIS-11 scores of students with and without IGD.

	**BIS**	**BAS-R**	**BAS-D**	**BAS-F**	**BIS-11**
	**M ±*SD***	**M ±*SD***	**M ±*SD***	**M ±*SD***	**M ±*SD***
IGD (*n* = 157)	15.34 ± 2.53	16.77 ± 2.32	11.23 ± 2.21	8.58 ± 2.00	71.7 ± 9.41
Non-IGD (*n* = 1,368)	14.27 ± 2.63	16.66 ± 2.36	11.22 ± 2.55	7.73 ± 1.77	64.1 ± 10.45
*T*	3.59^***^	0.41	0.02	3.96^***^	6.65^***^

*** *P < 0.001*.

### Comparison of BIS, BAS-R, BAS-D, BAS-F, and BIS-11 Among 157 Students of Different Ages With IGD

As can be seen in Table 2, each of the dimensions of BAS and impulsivity, exhibited no significant differences among the students of different ages with IGD. However, BIS scores of the college student group, were significantly higher than those of the high school and the middle school students (*p* < 0.05).

**Table 2:** BIS/BAS and BIS-11 scores of students with IGD in different age groups.

	**BIS**	**BAS-R**	**BAS-D**	**BAS-F**	**BIS-11**
	**M ±*SD***	**M ±*SD***	**M ±*SD***	**M ±*SD***	**M ±*SD***
Middle school student (*n* = 43)	19.63 ± 3.59	15.88 ± 2.65	10.95 ± 2.26	11.56 ± 2.23	70.95 ± 5.62
High school student (*n* = 48)	19.79 ± 3.11	16.08 ± 2.52	11.35 ± 1.95	10.94 ± 2.21	71.58 ± 5.48
College student (*n* = 66)	20.89 ± 2.29	16.76 ± 2.08	11.36 ± 2.33	11.70 ± 2.42	71.65 ± 5.11
*F*	3.10^*^	2.07	0.53	1.60	0.25
LSD	3>1,2				

* *p < 0.05, LSD3 > 1,2 means that college students have the highest score in the total score of the BIS scale, with a significant higher score compared to that of high school students, and a significant higher score compared to that of middle school students*.

### Correlation Analysis

The correlation between IAT score and BIS, BAS-R, BAS-D, BAS-F, and BIS-11 of 157 IGD students in different age stages was examined. There was not a significant correlation between IAT and BIS, each of the dimensions of BAS and BIS-11 among middle school students. There was a significant negative correlation between IAT and BAS-R among high school students (*r* = −0.28, *p* < 0.05). There was a significant positive correlation between IAT scores and BIS in college students (*r* = 0.375, *p* < 0.05).

### Roles of BAS/BIS and BIS-11 as Predictors of Addiction Among 157 Students of Different Ages With IGD

Regression analysis was conducted to test the roles of BIS, each of the dimensions of BAS and BIS-11 scores as predictors of IAT scores (Table 3).

**Table 3:** BIS/BAS regression analysis of adolescent Internet addiction in different periods.

**Model**	***R***	***R^**2**^***	**Adjusted *R^**2**^***	***F***
College group	0.464	0.215	0.149	3.284^*^
High school group	0.506	0.256	0.167	2.890^*^
Middle school group	0.178	0.032	−0.099	0.243

* *p < 0.05*.

The predictive variables of the college group model were BIS and BIS-11.

The predictive variables of the high school group model were BAS-R and BIS-F.

As can be seen from Table 3, the R2 model of the high school group was the larger, BIS and BIS-11 together could explain 14.9% variance of IGD among the college group, BAS-R and BAS-F together could explain 16.7% variance of IGD among the high school group. These two models demonstrated statistical significance (*p* < 0.05).

## Discussion

The primary objective of this study was to explore different risk factors for IGD among students of different ages. The BIS, each of the dimensions of BAS and impulsivity were compared between students with and without IGD by using the BIS/BAS questionnaire and the BIS-11. If we don't differentiate between ages, consistent with previous research, results indicated that there were significant differences between the IGD group and the non-IGD group in BIS, BAS-F, and impulsivity, with IGD showing elevated scores on these measures (13, 14, 19, 20).

Previous research has shown that BIS/BAS can change with age, with significant age effects observed for BIS and BAS-D (26). Similarly, the current study also found that the relationship between BIS/BAS and IAT is inconsistent among students with IGD of different age groups. For instance, The BIS scores of college students were highest of the three groups. Furthermore, analysis of the correlation between IAT scores and BIS/BAS, showed that IAT scores had a significant positive correlation with BIS among the college student IGD group, which indicated that college students had higher scores on BIS, and more likely that they suffered from IGD. We speculate those who score highly on BIS would experience more negative emotions, such as anxiety and depression, and may be more likely to escape into the virtual world of online games. Zhang (27) have also confirmed that College students' online game addiction is more related to emotion. Further regression analysis revealed that BIS and BIS-11 together could explain 14.9% variance of IGD among the college group. Mediating model found that impulsivity is directly related to online game addiction, and the effect of BIS on online game addiction works partly through impulsivity in our previous study (28). Additionally, IAT scores of high school students were significantly negative correlated with BASR. That is, the higher their BAS-R score, the less likely they are to have IGD, which means that getting more praise in the real world is a protective factor for IGD. Further regression analysis revealed that BAS-R and BAS-F together could explain 16.7% variance of IGD among the high school group. For high school students, the characteristics of network gaming itself might be one of causes leading to IGD. Internet games are extremely fun and meet the desire to achieve in the virtual world, especially for teenagers who are under learning pressure without enough leisure time and sport. But our study surprisingly found the IAT scores of middle school students were no correlated with BAS/BIS and impulsivity. We speculate that this difference is due to different psychopathological mechanisms of IGD at different ages. What's more, because our subjects excluded IGD patients with depression, we also did not examine the effect of mood on impulsivity, but control mechanisms might also depend on emotional factors (29).

The deficiency of this study lies in three point. Firstly, our sample size of IGD in different age groups was small; Secondly, the object of this study who is 157 IGD students of different ages which was 12–22 years, including adolescents and young adults here, so it is varied in the social and education environment, peer relationships, available leisure times; Last, the diagnostic criteria for IGD in DSM-5 have shortcomings, such as different item sensitivities, unclear cause-and-effect relationship with gaming, and difficulty in manipulating severity (30), which could have influenced the results. So we will add IGD subjects in different age groups, and explore the impact of impulsivity and BIS, each of the dimensions of BAS on IGD by incorporating social demographic characteristics and emotional state in future study.

## Conclusions

In the current study of students with IGD of different age, there were different possible psychological mechanisms contributing to the results. This suggests that further research should be carried out on patients with IGD in different age groups, so that more targeted interventions can be developed.

## Data Availability Statement

The raw data supporting the conclusions of this article will be made available by the authors, without undue reservation.

## Ethics Statement

The studies involving human participants were reviewed and approved by the Institutional Review Board at Guizhou Province People's Hospital. Written informed consent to participate in this study was provided by the participants' legal guardian/next of kin.

## Author Contributions

HX designed the study and wrote the protocol. XT, YZ, and JC conducted literature searches, provided summaries of previous research studies, and conducted the statistical analysis. MP provided revision of the manuscript. QZ edited and organized the manuscript. All authors contributed to and have approved the final manuscript.

## Conflict of Interest

The authors declare that the research was conducted in the absence of any commercial or financial relationships that could be construed as a potential conflict of interest.
